# Clinicopathologic features of endometrial cancer with mismatch repair deficiency

**DOI:** 10.3332/ecancer.2020.1061

**Published:** 2020-06-18

**Authors:** Sushmita Gordhandas, Ryan M Kahn, Charlotte Gamble, Nizam Talukdar, Brandon Maddy, Becky Baltich Nelson, Gulce Askin, Paul J Christos, Kevin Holcomb, Thomas A Caputo, Eloise Chapman-Davis, Melissa K Frey

**Affiliations:** 1 Department of Obstetrics and Gynecology, Weill Cornell Medical College, New York, NY, USA; 2 Division of Gynecologic Oncology, Department of Obstetrics and Gynecology, Weill Cornell Medical College, New York, NY, USA; 3 Department of Information Technologies and Services, Weill Cornell Medical College, New York, NY, USA; 4 Division of Biostatistics and Epidemiology, Department of Healthcare Policy & Research, Weill Cornell Medicine, New York, NY, USA

**Keywords:** microsatellite instability, mismatch repair endonuclease PMS2, MutS homolog 2 protein, EPCAM protein, human, epithelial cell adhesion molecule, immunohistochemistry, DNA mismatch repair, MLH1 protein, human, MutL protein homolog 1, neoplastic syndromes, hereditary, endometrial neoplasms, DNA, risk assessment

## Abstract

The inclusion of DNA mismatch repair (MMR) evaluation as a standard of care for endometrial cancer management will result in a growing population of patients with MMR deficiency and negative germline Lynch syndrome testing (MMR-deficient). In this systematic review and study, the clinicopathologic features of endometrial cancer in patients with MMR-intact, MLH1 methylation positive, MMR-deficient or Lynch syndrome are evaluated. A systematic search of online databases between 1990 and 2018 identified studies of endometrial cancer patients with tumour testing (MMR protein immunohistochemistry or microsatellite instability) and germline assessment for Lynch syndrome. Extracted data included tumour testing, germline genetic testing, age, body mass index (BMI), family history, tumour stage, grade and histologic type. Associations between MMR-intact, MLH1 methylation positive, MMR-deficient and Lynch syndrome groups were analysed using descriptive statistics. The comprehensive search produced 4,400 publications, 29 met inclusion criteria. A total of 7,057 endometrial cancer cases were identified, 1,612 with abnormal immunohistochemistry, 977 with microsatellite instability. Nine-hundred patients underwent germline genetic testing, identifying 212 patients with Lynch syndrome. Patients in the Lynch syndrome and MMR-deficient groups were significantly younger than patients in the MMR-intact and MLH1 methylation positive groups. Patients with MMR-intact tumours had the highest BMI, followed by MMR-deficient, then Lynch syndrome. MMR-intact tumours were more likely to be grade I at diagnosis than other groups. Patients with Lynch syndrome and MMR-deficient tumours were less likely to have stage I disease as compared to patients with MMR-intact tumours. Endometrial cancer patients with MMR-deficient tumours have similar features to those with germline Lynch syndrome mutations, including age, grade, histology and stage. Even in the absence of a germline mutation, tumour evaluation for MMR status may have important clinical implications.

## Introduction

In contrast to most solid tumours, the incidence and deaths from endometrial cancer continue to rise [1]. It is estimated that currently there are over 727,000 people in the United States living with endometrial cancer, with approximately 63,230 new cases and 11,315 deaths reported in 2018 [2, 3]. One proposed strategy to improve endometrial cancer outcomes is to abandon the traditional two-tiered endocrine based classification and move towards molecular tumour analysis [4]. Examples of such intiatives include The Cancer Genome Atlas (TCGA) and Proactive Molecular Risk Classifier for Endometrial Cancer (ProMisE) which all aim to identify prognostic signatures through specific molecular criteria [5, 6]. In 2014, the American College of Obstetricians and Gynecologists and Society of Gynecologic Oncology recommended that all endometrial tumours undergo molecular screening for mismatch repair (MMR) deficiency [7].

Evaluation of endometrial tumours for MMR deficiency can identify patients with Lynch syndrome, an autosomal dominant mutation in the DNA of MMR proteins (*MLH1, MSH2, MSH6 or PMS2* and *EPC*AM*).* Approximately 1 in 279 to 1 in 400 individuals is affected by Lynch syndrome and 3% of endometrial cancers are attributable to Lynch syndrome [8, 9]. The lifetime risk of endometrial cancer among patients with Lynch syndrome varies by gene: *MLH1* and *MSH2* 25%–60%, *MSH6* 16%–26%, and *PMS2* 15% [10]. MMR deficiency is typically evaluated via immunohistochemistry (IHC) for MMR protein expression, polymerase chain reaction assessment of DNA microsatellite instability (MSI) and *MLH1* promotor methylation analysis [11]. Based on a large meta-analysis, 28% of endometrial tumours will exhibit abnormal MMR IHC, and 31% have MSI. However, the majority of patients with abnormal MMR testing will not have an underlying Lynch syndrome mutation (15% of patients with abnormal IHC, and 19% with MSI) [9].

As more patients undergo MMR testing, there will be a growing population of patients found to have abnormal MMR testing and negative germline testing for Lynch syndrome [9]. This group will be referred to as MMR-deficient for the remainder of this paper. Recommendations from the National Comprehensive Cancer Network (NCCN) for the MMR-deficient group are vague, mentioning ‘tailored surveillance based on individual and family risk assessment’[10]. As endometrial cancer becomes a more heterogenous disease based on molecular profiling, an understanding of the pathologic and clinical implications of this stratification systems is imperative. Studies are beginning to find differences in outcomes between MMR-deficient, *MLH1* methylation positive, and MMR-intact endometrial cancers [12]. Furthermore, MMR status has a growing role in guiding management [13]. The aim of this study and systematic review is to evaluate the clinicopathologic features of patients with MMR-intact, *MLH1* methylation positive, MMR-deficient and Lynch syndrome endometrial cancers in order to better understand this expanding population.

## Methods

### Strategy for search and selection criteria

This systematic review was registered with PROSPERO (#84957), the international prospective register of systematic reviews, and followed the guidelines of the Preferred Reporting Items for Systematic Reviews and Meta-Analyses (PRISMA) statement [14]. This study provides additional analysis of data from a previously published meta-analysis reviewing tumour testing and diagnosis of Lynch syndrome [9].

A comprehensive literature search was conducted by the institutional medical librarian team on January 11, 2018 using the following bibliographic databases from inception: Ovid MEDLINE® (In-Process & Other Non-Indexed Citations and Ovid MEDLINE® 1946 to Present), Ovid EMBASE (1974 to present), and The Cochrane Library (Wiley). No language, publication date, or article type restrictions were included in the search (full Ovid MEDLINE search strategy is available in Supplementary Table 1). To be eligible, articles had to meet the following inclusion criteria: studies of patients with known endometrial cancer, studies whose patients underwent tumour testing with MMR IHC and/or MSI analysis, and studies whose patients underwent germline genetic testing for Lynch syndrome following positive screening. Exclusion criteria included: studies only regarding nonendometrial cancers, and studies whose subjects did not undergo genetic testing for Lynch syndrome. Both prospective and retrospective studies were included. The primary outcome was to describe the age, grade, stage, histology, and body mass index (BMI) of these groups using a systematic review approach.

### Data extraction

The number of patients with normal or abnormal IHC (for *MLH1, MSH2, MSH6* or *PMS2* genes), *MLH1* methylation, and MSI was extracted from each study if available (Supplementary Table 2). Data on age, BMI, family history criteria, tumour stage, tumour grade and histologic type were obtained when available (Supplementary Table 2). These variables were chosen because they were most commonly studied and reported.

Data were separated by MMR-intact, *MLH1* methylation positive, MMR-deficient or Lynch syndrome. MMR-intact included patients with normal MMR tumour testing (IHC and/or MSI). As noted previously, the MMR-deficient group included patients with abnormal MMR tumour testing and negative germline testing for Lynch syndrome mutations. Lynch syndrome included patients with abnormal MMR tumour testing and positive germline testing for Lynch syndrome mutations. Three independent investigators retrieved, entered, and exported data from the Covidence online software program.

### Statistical analysis

Associations between Lynch syndrome, MMR-deficient,* MLH1* methylation positive and MMR-intact groups were analysed using descriptive statistics. As presented in Supplementary Table 2, not all studies reported the clinicopathologic variables of interest. Studies that reported on any of the six variables were included in analysis.

The mean age and BMI were weighted based on the number of patients per study. Four studies had inclusion criteria of patients less than 50-year old, and therefore were excluded from calculation of mean age [15–18]. Only studies that reported a number of patients with a mean age/ BMI value were included in calculation of mean age and BMI for each group.

Associations between each cohort for continuous variables were conducted using *t*-test and analysis of variance. Post hoc (Tukey) tests were conducted between the four groups in analysis of variance testing and further analyzed using *t*-test for those that were statistically significant. For categorical variables, the four groups were analysed using chi-square. Test of significance was measured using two-tailed tests with a 95% acceptable error, *p*-value 0.05. All analyses were conducted with the use of Statistical Analysis System (SAS) software version 9.4 (SAS Institute).

## Results

### Search results

**Figure 1.**
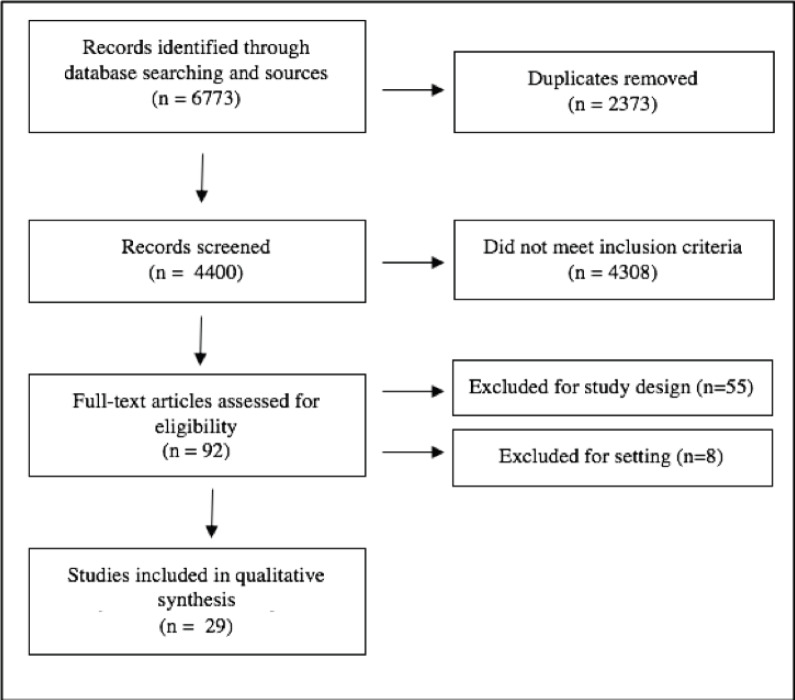
PRISMA flowchart of article search algorithm for systematic review.

A total of 6,773 studies were identified from the database search. All were imported into Covidence and duplicates were removed. The remaining 4,400 studies were screened by title and abstract against predetermined inclusion and exclusion criteria by three independent reviewers, with discrepancies resolved by consensus. Ninety-two articles were selected for full text review. Both reference and relevant article lists for these studies were gathered and duplicates were removed, producing 973 additional citations for review; none of these were selected for full text review. From the full text review, 29 articles met inclusion criteria (Figure 1, Supplementary Table 2) [15–43]. These studies were also described in a previously published meta-analysis reviewing tumour testing and diagnosis of Lynch syndrome [9].

### Patient characteristics

From the 29 included articles, 7,057 endometrial cancer cases were identified. Mean age of included patients was 59.7 years (range 35.7–64.6 years). Tumour histology included endometrioid (3,316, 86%), mixed (232, 6%), serous (166, 4%), carcinosarcoma (79, 2%), clear cell (36, 1%) and adenosquamous (16, 0.4%). Fifty-six percent of patients (2295) had grade 1 tumours, 26% (1062) grade 2 and 18% (736) grade 3. At time of diagnosis, 79% (2745) of patients were stage I, 6% (221) stage II, 13% (439) stage III and 2% (71) stage IV (Table 1).

Among the included patients, 6,325 (90%) underwent MMR tumour assessment via IHC and 3,140 (44%) via MSI analysis. Of the patients undergoing IHC, 1,612 had abnormal IHC staining for MMR proteins (1,162 *MLH1* absent, 450 *MSH2*, *MSH6* or *PMS2* absent). If *MLH1* was absent, *MLH1* promoter methylation was evaluated in the majority of studies (Supplementary Table 2). If known, *MLH1* promoter methylation positive tumours were not included in the MMR-deficient group. Of patients undergoing MSI analysis, 977 had MSI and 2,163 were microsatellite stable (Table 2).

Nine hundred patients underwent germline genetic testing, identifying 212 (24%) patients with Lynch syndrome. Six hundred eighty-eight (76%) patients were MMR-deficient (with negative germline mutation testing). Lynch syndrome was diagnosed in 3% of the total population and 13% of the population with abnormal tumour testing.

### Clinicopathologic findings

Five clinicopathologic variables were studied: age, grade, stage, histology and BMI at time of endometrial cancer diagnosis, presented in Table 3. For age at diagnosis, MMR-deficient and Lynch syndrome were similar (52.5 versus 51.4 years, respectively, *p* = 0.08). Patients with *MLH1* methylation positive tumours were significantly older than MMR-intact patients (64.2 versus 61.6 years, respectively, *p* < 0.01). Patients with Lynch syndrome and MMR-deficient tumours were significantly younger than patients with MMR-intact, and* MLH1* methylation positive tumours (*p* < 0.01).

Tumour grade was similar in MMR-deficient, *MLH1* methylation positive and Lynch syndrome. Patients with MMR-intact tumours were most likely to be diagnosed with low-grade disease, with 58% having FIGO grade I endometrial cancer. MMR-deficient, *MLH1* methylation positive and Lynch syndrome tumours were significantly less likely to be grade I than MMR-intact tumours (39% mismatch repair-deficient, 31% *MLH1* methylation positive, 41% Lynch syndrome (*p* < 0.01)).

Tumours with MMR-intact were significantly more likely to be endometroid histology than Lynch syndrome-associated tumours (87% versus 80%, respectively, *p* < 0.01). Ninety-one percent of *MLH1* methylation positive tumours were endometrioid histology, significantly more than MMR-intact and Lynch syndrome (*p* < 0.01). Stage at diagnosis was similar between MMR-deficient, and Lynch syndromes patients, both of these groups were significantly less likely to be diagnosed as stage I when compared to MMR-intact tumours (70% and 67% versus 81%, respectively, *p* < 0.01).

BMI was significantly different when comparing MMR-intact, MMR-deficient and Lynch syndrome cases. Patients with MMR-intact had the highest BMI (35.7 kg/m^2^), followed by MMR-deficient (34.6m kg/m^2^) and Lynch syndrome (27.6 kg/m^2^) (*p* < 0.01 for all comparisons).

Among the total population with available family history data, 30% met family history-based criteria for genetic assessment (Amsterdam and/or Bethesda criteria). MMR-deficient and MMR-intact patients were less likely to meet family history based criteria for germline testing than patients who were ultimately diagnosed with Lynch syndrome (13% and 19% versus 54%, *p* < 0.01) (Table 4). None of the studies evaluated BMI or family history among patients with *MLH1* methylation testing.

## Discussion

In this systematic review of 29 published studies and 7,057 patients, tumour testing in endometrial cancer helped classify groups of patients with similar clinicopathologic characteristics. The majority of patients with abnormal tumour MMR screening were negative for Lynch syndrome on germline testing (688/900, 76%), and therefore classified as MMR-deficient. We evaluated the clinicopathologic implications of this molecular finding with the hope of improving personalized disease management and patient counseling.

We found that MMR-deficient and Lynch syndrome groups were diagnosed with endometrial cancer at earlier ages compared to patients with MMR-intact (52.5 and 51.4 years versus 61.6 years respectively, *p* < 0.01). Younger age at diagnosis has been described in prior studies on MMR-deficient endometrial cancer [27]. Additionally, this reflects results seen in colorectal cancer literature where patients with Lynch syndrome and MMR-deficient tumours are also diagnosed at earlier ages than MMR-intact patients [27, 44].

Patients with Lynch syndrome were less likely to have endometrioid endometrial cancer than patients with MMR-intact tumours (80% versus 87%, *p* < 0.01). This reinforces previous literature showing that Lynch syndrome is associated with more heterogenous histologies [45]. Given the small sample size of patients with other histologies (serous, carcinosarcoma, clear cell and adenosquamous), no significant differences were identified amongst the cohorts.

Our data shows that BMI was highest among patients with MMR-intact tumours (35.7 kg/m^2^), followed by MMR-deficient and Lynch syndrome (32.6 and 27.6 kg/m^2^, respectively, *p* < 0.01). Therefore, obesity—an established risk factors for endometrial cancer—may not be associated with MMR defects. Previous endometrial cancer studies have reported mixed results when comparing BMI measurements between these groups [30, 46]. Our results, with a large sample size, suggest that genetic and epigenetic changes in MMR expression, rather than obesity, may drive the development of endometrial cancer in these populations.

The role of family history in genetic risk assessment is changing with growing utilisation of tumour molecular testing. There are various barriers that can inhibit obtaining an accurate family history. Because of this, family history-based testing is sensitive, but not specific, as 46% of patients in this study with Lynch syndrome did not have a family history that would warrant guideline-based genetic assessment. Only 13% of patients with MMR-deficient tumours met family history criteria. Patients with MMR-deficient tumours and MMR-intact tumours were less likely to meet family history criteria compared to those with Lynch syndrome (*p* < 0.01). This supports the understanding of MMR-deficiency as a sporadic change in the tumour that is not driven by germline mutations. This is also described in the colorectal cancer literature. In a 2013 observational cohort study of 1,705 colorectal cancer patients, Rodríguez–Soler *et al* [47] demonstrated the incidence of colorectal cancer in families of patients with MMR-deficient tumours was significantly lower than in families of patients with Lynch syndrome but higher than in families of patients with MMR-intact (*p* < 0.001).

In colorectal cancer, Lynch syndrome has been associated with lower stage and decreased risk of distant metastases at time of diagnosis when compared to the general population [48, 49]. However, in endometrial cancer we exhibited the opposite. Lynch syndrome and MMR-deficient endometrial cancers were less likely to present at stage I compared to patients with MMR-intact (*p* < 0.01).

The role of *MLH1* methylation as a prognostic factor is under evaluation. Current literature suggests that patients with *MLH1* promoter methylation have tumours that are larger, more deeply invasive, and likely to exhibit lympho-vascular space invasion compared to those without [12]. Cosgrove *et al* [12] suggests this could be explained by chemoresistance exhibited by MMR defective tumours with *MLH1* methylation. Our study shows that *MLH1* methylation positive, and MMR-deficient tumours were diagnosed at higher grade when compared to MMR-intact tumours. Additionally, patients with *MLH1* methylation positive tumours were diagnosed at a significantly older age than other groups.

To the author’s best knowledge, given the wide net of MMR-deficient patients included, this is one of the largest studies of patients with MMR-deficient endometrial cancer at this time. However, the MMR-deficient group may contain patients with unknown *MLH1* methylation status. *MLH1* methylation analysis and reporting varied between studies. MSI due to *MLH1* hypermethylation was not identified in studies that performed MSI analysis alone. Eleven studies did not report data on *MLH1* analysis, of which seven did not perform any *MLH1* methylation analysis (Supplementary Table 2) [15,16, 18, 19, 21, 33, 34, 37, 39, 40, 41].

Another limitation was the inconsistent reporting of clinicopathologic data and inclusion criteria amongst the different studies, resulting in a heterogeneous patient population. Because this was a retrospective systematic review, we were unable to control for subgroups amongst the cohorts which could have led to a bias of statistical findings. Additionally, most of the studies did not include follow up, therefore, data comparing clinical outcomes of MMR deficiency with and without Lynch syndrome are limited. Previous studies are mixed with some suggesting worse outcomes for patients with MMR defects, while others suggest improved or no difference in outcome [50–57]. In a meta-analysis from 2013, Diaz-Padilla *et al* [58] found no definitive evidence of a significant association between MMR status and survival in endometrial cancer.

On analysis of the TCGA subgroups (p53-mutant, MSI, *POLE*-mutant, and no specific molecular profile (NSMP)), prognosis was unfavourable in the p53- mutant group, intermediate in the MSI and NSMP group, and the *POLE*-mutant group had a favourable prognosis [6, 59].

In a recent publication by Backes et al, patients with high-intermediate risk endometrioid endometrial cancer and abnormal MMR expression had increased risk of recurrence and decreased recurrence-free survival compared with MMR-intact tumours [60]. In colorectal cancer, prior studies also demonstrate overall survival benefit with abnormal tumour testing [49].

Although the goal of tumour MMR screening was initially to identify patients at risk of carrying a Lynch syndrome mutation, we now see that tumour screening—even among Lynch syndrome negative patients—may have clinical implications. In May 2017, the US. Food and Drug Administration granted accelerated approval of Pembrolizumab for treatment of microsatellite-instability-high or deficient MMR, irrespective of tumour site or organ involved [13]. As a result, patients with MMR-deficient endometrial tumours now have access to indicated targeted therapy irrespective of Lynch syndrome status.

## Conclusions

This systematic review and study of the clinicopathologic features of endometrial cancers found that patients with MMR-deficient tumours have similar clinical features to those with germline Lynch syndrome which include age at diagnosis, grade, histology and stage. These findings will assist in patient counseling regarding results and interpretations of MMR testing. Further studies, including prospective designs, are necessary to better clarify the predictive and therapeutic implications of MMR-deficient endometrial cancers, and to help establish definitive management guidelines.

## Authors’ contributions

All authors have made substantial contributions to the conception/ design of the work, the acquisition, analysis, interpretation of data, the drafting of the work, and/or its critical revision for important intellectual content. All authors have given final approval of the version to be published.

## Conflicts of interest statement

The authors declare that they have no conflicts of interest.

## Funding declaration

Paul Christos, DrPH and Gulce Askin, MPH, were partially supported by the following grant: Clinical and Translational Science Center at Weill Cornell Medical College (1-UL1-TR002384-01).

## Figures and Tables

**Supplementary Table 1.** Full Ovid MEDLINE systematic review search strategy.

1. Lynch Syndrome II/	42. immunoenzymology.tw.
2. hereditary nonpolyposis colon cancer.tw.	43. antigen purification.tw.
3. hereditary non polyposis colon cancer.tw.	44. antigen isolation.tw.
4. hereditary nonpolyposis colorectal cancer.tw.	45. fluorescent antiglobulin*.tw.
5. hereditary non polyposis colorectal cancer.tw.	46. fluorescent inhibition.tw.
6. HNPCC.tw.	47. immunoassay/
7. (lynch adj3 syndome*).tw	48. immunoassay*.tw.
8. or/1-7	49. antibody assay*.tw.
9. DNA Methylation/	50. antibody test*.tw.
10. DNA Methylation*.tw.	51. antigen assay*.tw.
11. deoxyribonucleic acid methylation.tw.	52. immune assay*.tw.
12. DNA hypermethylation.tw.	53. immuno assay*.tw.
13. DNA hypomethylation.tw.	54. immunoanalysis.tw.
14. methylated deoxyribonucleic acid.tw.	55. enzyme linked immunosorbent assay*.tw.
15. methylated DNA.tw.	56. electroimmunoassay*.tw.
16. Methylation Analysis.tw.	57. immunospot assay*.tw.
17. exp Immunochemistry/	58. immunospot assay*.tw.
18. immunochemistry.tw.	59. immunoelectrophoresis.tw.
19. immunohistochemistry.tw.	60. immunoradiometric assay*.tw.
20. immunolabeling.tw.	61. interferon gamma release assay*.tw.
21. immunogold.tw.	62. radioimmunoassay*.tw
22. immunocytochemistry.tw.	63. immunoblotting/
23. fluorescent antibody technique/	64. immunoblot*.tw.
24. fluorescent antibod*.tw	65. immunocytochemistry.tw.
25. Immunofluorescence.tw.	66. cytoimmunochemistry.tw.
26. fluorescent protein tracing*.tw	67. immunocytochemical staining*.tw.
27. coon* techni*.tw	68. immunodiffusion/
28. fluorescent antinuclear antibody test*.tw	69. immunodiffusion.tw.
29. fluoroimmunoassay*.tw	70. antibody agar plate method*.tw.
30. immunofluorometric assay*.tw	71. immune diffusion.tw.
31. flourescence polarization immunoassay*.tw	72. immunoelectrodiffusion.tw.
32. antigen staining.tw.	73. ouchterlony*.tw.
33. immunostaining.tw.	74. immunoprecipitation/
34. antigen retrieval.tw.	75. immunoprecipitat*.tw.
35. immunoperoxidase staining.tw.	76. radionimmunoprecipitation.tw.
36. goodpasture staining*.tw	77. Microsatellite Instability/
37. peroxidase stain*.tw.	78. microsatellite Instability.tw.
38. immunoperoxidase technique*.tw.	79. (replication adj3 phenotype).tw.
39. immunophosphatase technique*.tw.	80. tumour typ*.tw.
40. immunochemical method*.tw.	81. or/9-80
41. immunochemical stud*.tw.	82. 8 and 81

**Supplementary Table 2.** Tumour testing performed, and studies including clinicopathologic data.

Study	Tumour testing				Age			BMI		
	IHC	MSI	*MLH1* Methylation	Tumour Sequencing	Intact	MMR-deficient	Lynch syndrome	Intact	MMR-deficient	Lynch syndrome
Adar Tomer 2018 [38]	X		X				X			
*Anagnostopoulos 2017* [15]	X	X	X						X	X
*Backes 2009* [39]	X				X					
*Banno 2003* [40]		X			X					
*Batte 2014* [41]	X		X				X			
*Berends 2003* [16]	X	X								
*Buchanan 2014* [42]	X		X				X			X
*Dillon 2017* [43]	X		X		X	X	X	X	X	X
*Dudley 2015* [19]	X									
*Egoavil 2013* [20]	X	X	X		X		X			
*Ferguson 2014* [21]	X	X				X	X		X	X
*Goodfellow 2015* [22]	X	X	X			X	X			
*Goodfellow 2003* [23]		X	X	X	X		X			
*Hampel 2006* [24]	X	X	X	X			X			
*Leenen 2012* [25]	X	X	X		X	X	X			
*Lin 2015* [26]	X		X			X	X			
*Lu 2007* [17]	X	X	X			X	X		X	X
*Mas-Moya 2015* [27]	X		X			X	X			
*Mills 2014* [28]	X		X							
*Mills 2016* [29]	X		X			X	X			
*Moline 2013* [30]	X	X	X		X	X	X	X		
*Najdawi 2017* [31]	X		X	X	X	X	X			
*Ollikainen 2005* [32]	X	X	X							
*Rubio 2016* [33]	X	X				X	X			
*Shin 2015* [34]	X	X			X		X			
*Sutter 2004* [18]	X	X		X	X					
*Watkins 2017* [35]	X		X							
*Westin 2008* [36]	X	X	X			X	X		X	X
*Yoon 2008* [37]	X	X	X							

Study	Stage			Grade			Histology		
	Intact	MMR-deficient	Lynch syndrome	Intact	MMR-deficient	Lynch syndrome	Intact	MMR-deficient	Lynch syndrome
Adar Tomer 2018 [38]									
*Anagnostopoulos 2017* [15]		X	X		X	X			
*Backes 2009* [39]	X	X		X	X		X	X	
*Banno 2003* [40]	X			X		X	X		
*Batte 2014* [41]			X			X			X
*Berends 2003* [16]									
*Buchanan 2014* [42]			X			X			x
*Dillon 2017* [43]	X	X	X	X	X	X	X	X	X
*Dudley 2015* [19]									
*Egoavil 2013* [20]				X			X		X
*Ferguson 2014* [21]			X			X			X
*Goodfellow 2015* [22]									
*Goodfellow 2003* [23]							X		X
*Hampel 2006* [24]									X
*Leenen 2012* [25]	X			X	X	X	X	X	X
*Lin 2015* [26]									
*Lu 2007* [17]		X	X					X	X
*Mas-Moya 2015* [27]					X	X		X	X
*Mills 2014* [28]									
*Mills 2016* [29]		X	X		X	X		X	X
*Moline 2013* [30]	X			X	X	X	X	X	X
*Najdawi 2017* [31]	X	X	X	X	X	X	X	X	X
*Ollikainen 2005* [32]									
*Rubio 2016* [33]	X	X	X						
*Shin 2015* [34]							X		X
*Sutter 2004* [18]	X			X			X		
*Watkins 2017* [35]									
*Westin 2008* [36]					X	X		X	X
*Yoon 2008* [37]									

X, study includes data in the specified population.

**Table 1.** Overall patient demographics and clinicopathologic findings.

Mean age- years (range)	59.7 (35.7–64.6)
**Tumour grade (n, %)**	
**1**	2,295 (56%)
**2**	1,062 (26%)
**3**	736 (18%)
**Tumour histology (n, %)**	
**Endometrioid**	3,316 (86%)
**Mixed**	232 (6%)
**Serous**	166 (4%)
**Carcinosarcoma**	79 (2%)
**Clear cell**	36 (1%)
**Adenosquamous**	16 (0.4%)
**Tumour Stage (n, %)**	
**I**	2,745 (79%)
**II**	221 (6%)
**III**	439 (13%)
**IV**	71 (2%)

**Table 2.** Molecular tumour testing results.

Population	*N*
Total population	7,057
Abnormal IHC	1,612
*MLH1* absent	1,162
*MLH1* methylation negative	166
*MLH1* methylation positive	996
*MSH2, MSH6,* or* PMS2* absent	450
Microsatellite insufficiency	977

**Table 3.** Clinicopathologic features of endometrial carcinomas with MMR-intact, MMR-deficient, MLH1 methylation positive and Lynch syndrome.

	MMR-intact	Abnormal tumour testing			P					
		MMR-deficient	*MLH1* methylation positive	Lynch syndrome	MMR-intact versus MMR-deficient	MMR-intact versus Lynch syndrome	MMR-deficient versus Lynch syndrome	MMR-intact vs. *MLH1* methylation positive	MMR-deficient vs. *MLH1* methylation positive	Lynch syndrome vs. *MLH1* methylation positive
Mean age- years (range)	61.6 (38.8–65.1)	52.5 (33.5–65.0)	64.2 (61.3–76.0	51.4 (44.0–57.9)	<0.01	<0.01	0.08	<0.01	<0.01	<0.01
Mean BMI- kg/m^2^ (range)	35.7 (34.4–37.0)	34.6 (31.0–35.1)		27.6 (23.8–30.3)	<0.01	<0.01	<0.01			
Grade (*n*, %)					<0.01	<0.01	0.89	<0.01	0.31	0.11
1	471 (58%)	31 (39%)	27 (31%)	39 (41%)						
2	175 (22%)	25 (32%)	37 (43%)	27 (28%)						
3	165 (20%)	23 (29%)	22 (26%)	29 (31%)						
Histology (*n*, %)					0.06	<0.01	0.06	<0.01	0.44	<0.01
Endometrioid	979 (87%)	128 (92%)	248 (91%)	107 (80%)						
Mixed	43 (4%)	7 (5%)	14 (5%)	13 (10%)						
Serous	76 (7%)	1 (1%)	3 (1%)	4 (3%)						
Carcinosarcoma	14 (1%)	0 (0%)	3 (1%)	0 (0%)						
Clear cell	13 (1%)	2 (1%)	1 (0.4%)	4 (3%)						
Adenosquamous	4 (0.4%)	1 (0.7%)	3 (1%)	5 (4%)						
Stage (*n*, %)					<0.01	<0.01	0.76	0.28	0.17	0.12
I	608 (81%)	130 (70%)	64 (73%)	56 (67%)						
II	49 (7%)	19 (10%)	9 (10%)	10 (12%)						
III	69 (9%)	36 (19%)	11 (12%)	17 (21%)						
IV	22 (3%)	2 (1%)	4 (5%)	0 (0%)						

MMR, DNA mismatch repair; BMI, body mass index

**Table 4.** Family history based criteria* for germline testing with MMR-intact, MMR-deficient and Lynch syndrome.

		Overall (*N* = 456)	MMR-intact (*N* = 146)	Abnormal tumour testing		*p*		
				MMR-deficient (*N* = 148)	Lynch syndrome (*N* = 162)	MMR-intact versus MMR-deficient	MMR-intact versus Lynch syndrome	MMR-deficient versus Lynch syndrome
Family history*	Yes	135 (30%)	28 (19%)	19 (13%)	88 (54%)	0.13	<0.01	<0.01
	No	321 (70%)	118 (81%)	129 (87%)	74 (46%)			

* Family history based criteria defined as Amsterdam and/or Bethesda criteria, per study.
